# Predictors and Biomarkers of Subclinical Leaflet Thrombosis after Transcatheter Aortic Valve Implantation

**DOI:** 10.3390/jcm9113742

**Published:** 2020-11-21

**Authors:** Katarzyna Pieniak, Szymon Jędrzejczyk, Olaf Domaszk, Kajetan Grodecki, Bartosz Rymuza, Zenon Huczek, Janusz Kochman, Krzysztof J. Filipiak, Aleksandra Gąsecka

**Affiliations:** 1 Department of Cardiology, Medical University of Warsaw, 02-091 Warsaw, Poland; katarzyna.m.pieniak@gmail.com (K.P.); s.jedrzejczyk64@gmail.com (S.J.); olafdomaszk@o2.pl (O.D.); kajetan.grodecki@gmail.com (K.G.); bartosz.rymuza@gmail.com (B.R.); zhuczek@wp.pl (Z.H.); jkochman@wum.edu.pl (J.K.); krzysztof.filipiak@wum.edu.pl (K.J.F.); 2 Laboratory of Experimental Clinical Chemistry, Academic Medical Centre, Amsterdam University Medical Centres, 1105 AZ Amsterdam, The Netherlands

**Keywords:** leaflet thrombosis, predictors, biomarkers, aortic stenosis, TAVI

## Abstract

Transcatheter aortic valve implantation (TAVI) is a recent revolutionary treatment for high-risk patients with severe aortic stenosis who are not suitable for surgery, expanding to intermediate and low-risk patients. Valve leaflet thrombosis (LT) is a potentially fatal complication after TAVI. The incidence of subclinical LT is as high as 25% among patients in the first year after TAVI. Subclinical LT may evolve into symptomatic thrombosis or lead to premature bioprosthesis degeneration, increasing the risk of neurological complications. Because imaging-based methods have limited sensitivity to detect subclinical LT, there is an urgent need for predictors and biomarkers that would make it possible to predict LT after TAVI. Here, we summarize recent data regarding (i) patient-related, (ii) procedure-related, (iii) blood-based and (iv) imaging predictors and biomarkers which might be useful for the early diagnosis of subclinical LT after TAVI. Prevention of LT might offer an opportunity to improve risk stratification and tailor therapy after TAVI.

## 1. Introduction

Aortic stenosis (AS) is the most common valvular heart disease in Europe and North America, with increasing prevalence due to the aging of the population. When untreated, aortic stenosis confers poor survival, with a 5-year mortality rate of 56% in moderate stages and 67% in severe stages [1]. The interventional treatment in symptomatic AS is either surgical aortic valve replacement (SAVR) or percutaneous transcatheter aortic valve implantation (TAVI). While designed to treat severe aortic stenosis in patients at high surgical risk, TAVI has recently been recognized as an alternative treatment strategy in patients at moderate and low risk [2]. Based on the results of randomized studies comparing the safety and efficacy of both methods, TAVI was noninferior to SAVR with respect to death from any cause or disabling stroke in a low-risk population at two-year follow-up [3]. The most common nonaccess site-related complications of TAVI include atrioventricular and intraventricular conduction disturbances and subclinical leaflet thrombosis (LT) [4,5,6]. Whereas conduction disturbances are relatively easy to treat with pacemaker implantation, LT is difficult to diagnose and treat, and has potentially fatal complications. The incidence of clinically symptomatic LT is low (reported in 0.5% of patients per year), but subclinical LT is much more common [7,8]. Subclinical LT is detected on multidetector computed tomography (MDCT) imaging as a hypo-attenuating leaflet thickening (HALT), which is a thin layer of thrombus covering one or more leaflets of the aortic valve [9]. In the recent PARTNER 3 (The Safety and Effectiveness of the SAPIEN 3 Transcatheter Heart Valve in Low-Risk Patients With Aortic Stenosis) CT substudy, where 435 patients with low-surgical-risk aortic stenosis were randomized to TAVI (*n* = 221) or SAVR (*n* = 214), the incidence of HALT increased from 10% at 30 days to 24% at one year [10]. Spontaneous resolution of 30-day HALT occurred in 54% of patients at one year, whereas new HALT appeared in 21% of patients at this time. The impact of HALT on thromboembolic complications and structural valve degeneration needs further assessment. It is believed that subclinical LT may lead to the increased transvalvular gradients and evolve into symptomatic thrombosis [7]. It may also be responsible for the premature bioprosthesis degeneration and increase the risk of neurological complications, such as stroke or transient ischemic attack [11]. Hence, there is an urgent need for predictors and biomarkers that would make it possible to predict LT after TAVI, or to diagnose it at an early stage. Finding such predictors and biomarkers could accelerate LT diagnoses, prevent subsequent adverse cardiovascular events, and finally, allow us to tailor treatments (anticoagulation). Here, we summarize recent data regarding (i) patient-related, (ii) procedure-related, (iii) blood-based, and (iv) imaging predictors and biomarkers which might potentially be useful to predict and/or diagnose subclinical LT after TAVI. These predictors and biomarkers are summarized in Table 1.

## 2. Patient-Related Predictors

### 2.1. Gender 

Subclinical LT is usually detected on MDCT imaging as a HALT, which is a thin layer of thrombus covering one or more leaflets of the aortic valve [9]. The increased incidence of HALT is associated with male sex [12]. Although the reason for this association remains unclear, the increased risk of LT in male patients may be potentially explained by procedural and anatomical differences. Men are more likely to receive large prosthetic valves [23]. For example, in a study encompassing 405 patients who underwent MDCT 1–3 months after TAVI, the valve size positively correlated with the risk of LT. Whereas no LT was observed in patients with 23 mm prosthesis, the prevalence of LT was increased in patients with the prosthesis size of 26 and 29 mm (6.1% and 11.8%, respectively) [16]. Another reason for the increased risk of LT in male patients may be a larger mean diameter of sinus of Valsalva, compared to women (31.6 mm vs. 27.2 mm, respectively) [24]. Since TAVI is associated with a significant decrease in blood flow velocity in the sinuses of Valsalva, larger diameter may predispose to blood retention, with increased risk of thrombus formation [15,25].

### 2.2. Comorbidities

The comorbidities associated with increased risk of LT after TAVI include (i) obesity, (ii) hypertension, (iii) chronic obstructive pulmonary disease (COPD) and (iv) smoking history.

Obesity is an independent predictor of LT after TAVI [26]. Patients with a body mass index of more than 30 kg/m^2^ are more predisposed to arterial thrombosis because of chronic inflammation and an imbalance between the concentrations of pro- and anti- thrombotic molecules. For instance, obese patients have decreased expression of microRNA-126, which has antithrombotic effects by preventing platelet-endothelial cell interactions. Further, increased risk of LT in obese patients may be due to the increased level of prothrombotic and antifibrinolytic molecules (resistin, visfatin, plasminogen activator inhibitor-1), as well as decreased plasma level of antithrombotic adiponectin [26].

Another risk factor of LT is hypertension. In a retrospective study including 138 patients, HALT and leaflet motion limitations were already observed more often in hypertensive patients 17.5 days after valve implantation [27]. In patients with chronically elevated blood pressure, cardiac output gradually decreases due to the increased total peripheral resistance and left ventricular hypertrophy, and reduced blood flow through the aortic valve seems to increase the risk of LT.

In another study including 170 patients with Sapien 3 valves, HALT was observed in 16% of patients at 30 days; its occurrence correlated with the history of COPD [27]. Hypercoagulable state in COPD may be explained by the constant bronchial and systemic inflammation. Moreover, chronic hypoxia leads to changes in platelet membrane and activation of cyclooxygenase-1 and thromboxane formation, increasing platelet reactivity [13].

Concurrently, smoking history is another risk factor of systemic inflammation which contributes to LT [28]. The mechanism of smoking as a factor increasing the risk of arterial thrombosis is complex. Endothelial cell injury, platelet dysfunction and increased concentrations of fibrinogen and coagulation factors are all associated with the risk of thrombus initiation and propagation [29].

The risk of LT is lower in patients with atrial fibrillation (AF), which is likely associated with oral anticoagulation (OAC) treatment [30]. In a meta-analysis including eighteen studies encompassing 11,124 patients, patients treated with OAC were less likely to develop LT at six-month follow-up, whereas single antiplatelet therapy was an independent risk factor of LT [12]. Since platelet activation is a prerequisite for thrombus formation, the increased risk of LT associated with single antiplatelet therapy may be due to insufficient platelet inhibition in this group of patients. Hence, in patients who do not need OAC, DAPT should be considered for the first 3–6 months after TAVI, followed by lifelong single antiplatelet therapy [31]. On the other hand, the results of recent trials support the use of aspirin alone to minimize the bleeding risk without increasing the ischaemic risk after TAVI [32,33]. However, the subclinical LT was not evaluated in these studies. Altogether, the optimal antiplatelet therapy regimen after TAVI remains disputable and certainly requires a balance between the risk of thrombotic and bleeding events.

Nonvitamin K oral anticoagulants (NOAC) seem to be comparable to vitamin K antagonists in terms of stroke prevention and bleeding after TAVI, although the data about the efficacy of dabigatran, rivaroxaban, apixaban and edoxaban in LT prevention remain limited and require further investigation [14,22]. In a substudy of the GALILEO (Global Study Comparing a Rivaroxaban-based Antithrombotic Strategy to an Antiplatelet-based Strategy after Transcatheter Aortic Valve Replacement to Optimize Clinical Outcomes) trial involving 231 patients without indication of long-term anticoagulation, a rivaroxaban-based antithrombotic strategy was more effective than an antiplatelet-based strategy in preventing subclinical leaflet-motion abnormalities at a mean 90 ± 15 days after randomization [34]. For example, thickening of at least one leaflet was observed in 12 of 97 patients (12.4%) in the rivaroxaban group, and in 33 of 102 (32.4%) in the antiplatelet group (difference: −20.0 percentage points; 95% confidence interval (CI): −30.9 to −8.5) [34]. However, the rivaroxaban-based strategy was associated with a higher risk of death or thromboembolic complications and a higher risk of bleeding than the antiplatelet-based strategy [35]. Hence, although OAC (rivaroxaban) treatment decreases the risk of LT, it may deteriorate the prognosis, leaving no consensus regarding the optimal treatment regimen to prevent and treat subclinical LT after TAVI.

## 3. Procedure-Related Predictors

**Figure 1:**
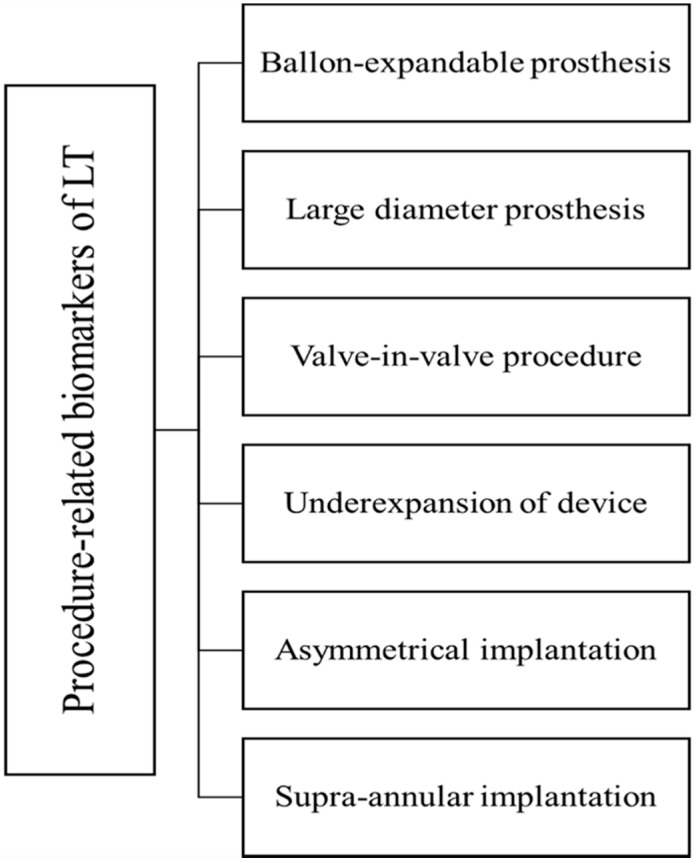
Procedural characteristics predisposing to leaflet thrombosis (LT) after transcatheter aortic valve implantation.

The currently known procedure-related predictors of LT in transcatheter heart valves (THVs) include (i) underexpansion and asymmetrical implantation of the valve, (ii) balloon-expandable prosthesis, (iii) large-diameter prosthesis, (iv) supra-annular implantation and (v) valve-in-valve procedure (ViV-TAVI). The procedural characteristics predisposing to LT after TAVI are summarized in Figure 1.

A recent study showed an association between underexpansion of THVs and probability of LT [17]. The SAPIEN 3 valves were expanded to three diameters, corresponding to 80%, 90% and complete expansion. Complete expansion resulted in the decrease in blood residence time on the surface of the leaflets, in comparison to 80% and 90% THV expansion. There was a positive, linear correlation between the area of blood stasis time and the degree of underexpansion [17]. Although the increased blood residence time does not lead to thrombogenesis by itself, incomplete expansion can create leaflet folds and potential recesses for thrombus formation [36]. On the other hand, aggressive postdilatation may damage the prosthesis’s leaflets and subsequently increase the risk of LT [36]. Increased risk of THV thrombosis might be also related to asymmetrical implantation of the valve [37]. However, other studies did not show that procedural repositioning, postdeployment THV geometry or degree of THV oversizing increased the incidence of LT [16,38,39,40]. Altogether, it seems that symmetrical implantation and complete THV expansion may lower the risk of LT, but this issue requires further investigation.

An observational single-center study reported 7% incidence of HALT in a group of 405 consecutive patient who underwent TAVI with balloon-expandable Edwards Sapien XT or Sapien 3 [16]. The increased risk of LT was observed in the group that required treatment with larger THV (*p* = 0.03), and multivariable analysis showed that 29 mm THV is an independent predictor of LT (relative risk (RR): 2.89; 95% CI: 1.44 to 5.80) [16]. A similar finding was reported in another study [15]. In a recent meta-analysis, large diameter prostheses, which are commonly used in overweight patients, were found to be one of the most relevant predictors for LT, which could be explained by the reduced transvalvular flow velocity in larger THV with subsequent blood stasis [11]. In addition, the risk of underexpansion of THV correlated with the large-diameter prosthesis in obese patients [41,42].

LT can occur both in self-expandable and balloon-expandable devices. Results from the RESOLVE/SAVORY registries suggest that the difference in the rates of LT between valve types correlates with the supra-annular versus intra-annular design, rather than the THV type [7]. However, the intra-annular deployment, typical for balloon-expandable devices, was shown to be an independent predictor of LT in a recent meta-analysis [11]. The possible mechanism underlying this correlation might be related to the balloon-dilatation-induced tissue injury. Also, the leaflets of the native valve may overlap with the balloon-expandable THV, contributing to blood stasis, platelet activation and thrombus formation.

Finally, it was shown that patients with ViV TAVI are more likely to experience LT [18,37], especially if the prosthesis is implanted in stented porcine valve platform [18,43,44]. Although the precise mechanism behind this association is not clear, the available evidence suggests local hemodynamic derangement after ViV procedures [45,46,47]. For example, blood residence time is increased on the leaflets of ViV implanted device, and the absence of endothelial cells on the valve combined with tissue damage from ViV-TAVI insertion might increase the procoagulant state [48]. Further studies are needed to fully understand the procedural factors contributing to the increased risk of LT, especially in patients with moderate and low surgical risk.

## 4. Blood-Based Biomarkers

### 4.1. Markers of Coagulation and Fibrinolysis

Markers of coagulation and fibrinolysis, including von Willebrand factor (VWF), thrombin-antithrombin complex (TAT), plasmin-α_2_-antiplasmin complex (PAP), prothrombin activation fragment 1 + 2 (F1 + 2) and D-dimer, have been used to detect thrombus formation in numerous studies. VWF plays a major role in primary hemostasis by promoting the adhesion of platelets to subendothelial collagen at sites of vascular damage and platelet aggregation [49]. TAT is composed of thrombin and its inhibitor, and is measured as an alternative to thrombin due to thrombin instability. PAP is a complex of plasmin and α_2_-antiplasmin. The presence of PAP in plasma is a direct indicator of hypofibrinolytic state, whereas decreased PAP levels predict the perioperative venous thromboembolism (VTE) [50]. Prothrombin fragments F1 + 2 are produced during the transition of prothrombin into thrombin [51]. *D*-dimer is a fibrin degradation product of high sensitivity for ongoing thrombotic processes including VTE, but low specificity [52].

In a recent study comprising 43 patients, altered VWF collagen-binding capacity was observed in nearly 30% of patients after TAVI, but not after SAVR, which may indicate differences in the response of the hemostatic system to THV and operation [53]. However, the association between WVF and subclinical LT has not yet been established. The concentrations of TAT, PAP, prothrombin activation F1 + 2 and D-dimer were assessed in a clinical trial including 307 patients undergoing transfemoral TAVI preoperatively and up to day seven after the intervention [19]. Among the studied biomarkers, the concentrations of TAT, PAP and D-dimer exceeded the normal range prior to TAVI, and the concentrations of TAT, PAP, D-dimer and F1+2 increased immediately after TAVI [19]. However, none of these biomarkers correlated with LT, likely due to their low specificity. Another reason could be that the studies performed so far included a rather low number of patients, and larger studies could be more informative. 

### 4.2. Natriuretic Peptides

The concentrations of natriuretic peptides (NPs) in serum are elevated in numerous cardiovascular pathologies including cardiac hypertrophy, heart failure, acute coronary syndromes and AS. In the study including 333 patients, changes in NT-proBNP concentrations were serially evaluated from baseline to 24 months after TAVI [20]. The majority of patients undergoing TAVI presented with the elevated concentration of NT-proBNP at baseline, and higher NT-proBNP concentration was associated with greater overall and cardiac mortality at a median follow-up of 24 months [20]. In another study, the predictive value of NT-proBNP was evaluated in 504 patients with normal left ventricle ejection fraction (LVEF) undergoing TAVI, and was shown to correlate with patient survival [54]. Hence, patients with normal LVEF and distinctly elevated NT-proBNP concentration seem to have increased risk of mortality after TAVI [54]. Nevertheless, these studies did not prove the correlation between the NT-proBNP concentration and the risk of LT. 

In a study including 642 patients who underwent TAVI, the median time required to diagnose clinical LT was 181 days (interquartile range: 25 to 297 days) [18]. NT-proBNP plasma concentrations were increased in patients who were symptomatic or had elevated transvalvular gradients during follow-up and were eventually diagnosed with LT [18]. Moreover, NT-proBNP was reduced after VKA therapy, which was administered to all patients who were diagnosed with THV thrombosis [18]. Hence, despite the facts that NT-proBNP is a very unspecific biomarker and suspicion of LT after TAVI cannot be based solely on the NT-proBNP concentration, NT-proBNP could potentially be used to monitor the regression of LT during OAC treatment.

### 4.3. Platelet Extracellular Vesicles (EVs)

**Figure 2:**
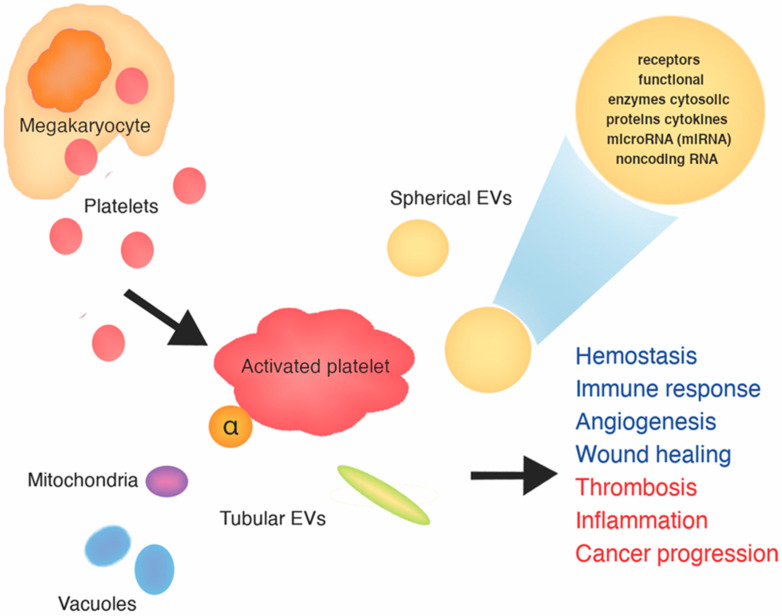
Platelet-based mechanisms which might potentially underlie leaflet thrombosis after transcatheter aortic valve implantation.

Platelets are anuclear fragments of megakaryocytes involved in hemostasis, immune response, angiogenesis and wound healing. On the other hand, platelets contribute to thrombosis, inflammation and cancer progression [55]. Because activated platelets release numerous mediators from their α-granules, secrete pro-inflammatory organelles like mitochondria and vacuoles and release extracellular vesicles (EVs), indicators of platelet activation have been studied as potential biomarkers of thrombosis [55]. The platelet-based mechanisms which might potentially underlie LT after TAVI are presented in Figure 2.

EVs are nanoparticles released by different cell types to body fluids. Due to the association between EVs and thrombosis, EVs drew the attention of researchers looking for markers of LT after TAVI [56]. EVs contain lipids, proteins, nucleic acids including micro-RNA and noncoding RNA, thereby mediating the intercellular communication [57]. In pathological conditions, including thrombosis, EVs may serve as modulators of inflammation, vascular dysfunction and thrombosis [56]. Due to the key role of platelets in thrombosis, platelet-derived EVs are of particular interest. EVs participate in thrombus formation due to phosphatidylserine (PS) and tissue factor (TF) exposure [58]. PS provides the surface for the assembly of the coagulation factors, therefore supporting thrombin generation, whereas TF is the most potent clotting activator in vivo [58]. Circulating EVs contribute to thrombosis also through indirect mechanisms, by enhancing platelet adhesion to collagen-coated surfaces and atherosclerotic plaques via P-selectin [59]. It has been suggested that EVs may be ~100-fold more procoagulant than activated platelets [21]. It is noteworthy that the TAVI procedure was shown to modulate the composition of EVs in the bloodstream by decreasing the concentration of platelet EVs and increasing the concentration of EVs from endothelial cells [60]. The variety of EVs, their contents and functions hold promise as potential molecules specific to LT. However, whether EVs are potential biomarkers of LT after TAVI requires further investigation.

## 5. Imaging Predictors

The imaging of patients referred for TAVI is a comprehensive and multimodal process, routinely combining (i) echocardiographic and (ii) computed tomography (CT) evaluation. Both modalities may be potentially used to identify patients at risk of developing LT.

### 5.1. Echocardiographic Predictors

Echocardiography plays a key role in diagnosing AS, TAVI guidance, and postprocedural monitoring. While echocardiographic identification of low-flow conditions is necessary to ensure prompt intervention, the impaired hemodynamical status might also favor LT [61]. The role of decreased cardiac output in promoting the formation of thrombi was initially confirmed in a combined analysis of RESOLVE and SAVORY registries, where low EF was identified as an independent predictor of LT, confirmed with MDCT in a mixed population of surgical and transcatheter patients up to one year after the procedure [7]. Moreover, recent results from a multicenter OCEAN-TAVI Registry showed that low-flow low-gradient AS increases the risk of early LT (detected in MDCT at the median of three days after TAVI) in patients receiving balloon-expandable valves nearly three-fold [38]. In the same registry, the risk of late LT (detected 30 days after TAVI) was found to increase two-fold following the in-hospital identification of less than mild paravalvular leak. These observations underline the importance of hemodynamic stasis on the leaflets in LT development [38,62]. On the other hand, none of these studies has demonstrated an association between the baseline or predischarge echocardiographic indices and the presence of LT [12,13,15,63]. Substantial variability in echocardiographic findings may reflect contemporary differences in patient characteristics, implantation techniques, and finally, the timing or intensity of LT screening [7,13,38].

### 5.2. Computed Tomography Predictors

Given its superior spatial resolution compared to echocardiography, CT remains the gold standard for LT detection. Although CT is widely used in TAVI procedural planning, there is a paucity of data on the CT-associated predictors of LT. Preprocedural CT enables a complete characterization of the device landing zone using quantitative techniques with the application of Hounsfield Unit (HU) thresholds [64]. A typical work-up in TAVI patients involves the assessment of the calcified tissue deposits that could contribute to LT by restricting expansion and/or changing the geometry of the THV. However, additional quantification of noncalcified tissue might extend our knowledge of the role of valve composition in LT, as it is hypothesized that the damaged native valve tissue may induce thrombosis due to exposure of TF [9,65]. Until now, the association between noncalcified coronary plaque subsets with adverse cardiac events, as well as unfavorable procedural outcomes of percutaneous coronary interventions, have been well evidenced [66,67]. The anatomy of the aortic valve complex also does not seem to effect the LT risk, with only one study demonstrating an association between the larger diameter of Valsalva sinuses and HALT [15]. 

The quantitative features of periaortic adipose tissue are another promising metric with which to identify patients at risk of developing LT. Both periaortic adipose tissue volume and attenuation may serve as markers of inflammation, triggered by the endothelial injury of the device landing zone following THV implantation [68]. The bidirectional interplay between adipose tissue and the coronary vascular wall has been extensively studied in the past, thus providing a validated methodology ready to be adapted into LT research [69].

Ultimately, the amount of quantitative information accessible from CT images may be greatly increased with the radiomic approach, allowing for the extraction of multiple imaging features which are indiscernible to the human eye [70]. While traditional quantitative analyses simply enumerate the average voxel intensity values without considering the spatial relationship between them, radiomics utilizes texture analysis to model the spatial distribution of voxel grey-level intensities, applies statistics to provide a measure of heterogeneity and quantifies the size and shape of three-dimensional volumes within an imaging dataset [71]. Such refined extraction of imaging details from a region of interest may create big data patterns associated with LT.

## 6. Conclusions

There is a wide spectrum of potential predictors and biomarkers of LT after TAVI (Table 1). Patient’s characteristics including male sex and comorbidities (hypertension, COPD, obesity, smoking history) are associated with the increased risk of LT after TAVI. In contrast, patients with AF are less likely to develop LT, which might be associated with chronic OAC therapy. Procedure-related predictors of LT include underexpansion and asymmetrical implantation of the device, large-diameter prosthesis, balloon-expandable prosthesis, supra-annular implantation and ViV-TAVI. Hence, complete expansion and symmetrical implantation of THV, along with minimizing intraprocedural manipulations, are desirable to reduce the risk of LT. Blood-based biomarkers could enable minimally invasive LT diagnoses. Higher concentrations of NT-proBNP, TAT, PAP, D-dimer or F1 + 2 are present in patients with LT after TAVI. Nevertheless, none of them is specific enough to predict LT. Regarding the association between EVs and thrombosis, platelet-derived EVs could become a promising biomarker of LT, but this requires further investigation. At present, solely imaging techniques can be used to identify patients at risk of developing LT, with MDCT remaining the gold standard for LT detection.

## Figures and Tables

**Table 1:** Potential predictors and biomarkers of leaflet thrombosis (LT) after transcatheter aortic valve implantation. All endpoints refer to sublinical LT.

Predictor or Biomarker	Design	Group	Endpoint	Ref
**Patient-based**				
Atrial fibrillation	Prospective, single-center	N = 754	↓ LT	[12]
COPD	Prospective, single-center	N = 170	↑ LT	[13]
Male sex	Prospective, single-center	N = 754	↑ LT	[12]
NOAC treatment	Retrospective, single-center	N = 154	↓ LT	[14]
OAC treatment	Meta-analysis	N = 11,124	↓ LT	[11]
Obesity	Meta-analysis	N = 11,124	↑ LT	[11]
Smoking	Prospective, single-center	N = 170	↑ LT	[13]
**Procedure-based**				
Large diameter THV	Prospective, single-center	N = 70	↑ LT	[15]
Large diameter THV	Prospective, single-centeral	N = 405	↑ LT	[16]
Supra-annular implantation	Registry	N = 890	↑ LT	[7]
Underexpansion	Computational and experimental (porcine valve platform)	–	↑ LT	[17]
ViV procedure	Retrospective, single-center	N = 642	↑ LT	[18]
**Blood-based**				
TAT, PAP, prothrombin activation fragment 1 + 2, D-dimer	Prospective, single-center	N = 307	↑ post-TAVI prothrombotic state	[19]
NT-proBNP	Prospective, multi-center	N = 333	↑ mortality, ↑ heart failure, ↑ atrial fibrillation	[20]
Platelet EVs	Experimental study	–	↑ arterial thrombosis	[21]
**Imaging**				
Low-flow low-gradient AS	Prospective, multi-center	N = 485	↑ early LT (3 days after TAVI)	[22]
Paravalvular leak	Prospective, multi-center	N = 485	↑ late LT (30 days after TAVI)	[22]
Reduced EF	Prospective, single-center	N = 890	↑ LT (2 weeks after SAVR, 4 weeks after TAVI)	[7]

Abbreviations: ↑: increased frequency; ↓: decreased frequency; AS: aortic stenosis; COPD: chronic obstructive pulmonary disease; EF: ejection fraction; EVs: extracellular vesicles; LT: leaflet thrombosis; NOAC: novel oral anticoagulants; NT-proBNP: N-terminal pro B-type natriuretic peptide; OAC: oral anticoagulants; PAP: plasmin-α_2_-antiplasmin complex; TAT: thrombin-antithrombin complex; TAVI: transcatheter aortic valve implantation; THV: transcatheter heart valve; ViV: valve-in-valve.
